# Alpha-Toxin Induces Programmed Cell Death of Human T cells, B cells, and Monocytes during USA300 Infection

**DOI:** 10.1371/journal.pone.0036532

**Published:** 2012-05-04

**Authors:** Tyler K. Nygaard, Kyler B. Pallister, Ashley L. DuMont, Mark DeWald, Robert L. Watkins, Erik Q. Pallister, Cheryl Malone, Shannon Griffith, Alexander R. Horswill, Victor J. Torres, Jovanka M. Voyich

**Affiliations:** 1 Department of Immunology and Infectious Diseases, Montana State University – Bozeman, Montana, United States of America; 2 Department of Microbiology, New York University School of Medicine, New York, New York, United States of America; 3 Department of Microbiology University of Iowa, Iowa City, Iowa, United States of America; National Institutes of Health, United States of America

## Abstract

This investigation examines the influence of alpha-toxin (Hla) during USA300 infection of human leukocytes. Survival of an USA300 isogenic deletion mutant of *hla* (USA300Δ*hla*) in human blood was comparable to the parental wild-type strain and polymorphonuclear leukocyte (PMN) plasma membrane permeability caused by USA300 did not require Hla. Flow cytometry analysis of peripheral blood mononuclear cells (PBMCs) following infection by USA300, USA300Δ*hla*, and USA300Δ*hla* transformed with a plasmid over-expressing Hla (USA300Δ*hla* Comp) demonstrated this toxin plays a significant role inducing plasma membrane permeability of CD14^+^, CD3^+^, and CD19^+^ PBMCs. Rapid plasma membrane permeability independent of Hla was observed for PMNs, CD14^+^ and CD19^+^ PBMCs following intoxication with USA300 supernatant while the majority of CD3^+^ PBMC plasma membrane permeability induced by USA300 required Hla. Addition of recombinant Hla to USA300Δ*hla* supernatant rescued CD3^+^ and CD19^+^ PBMC plasma membrane permeability generated by USA300 supernatant. An observed delay in plasma membrane permeability caused by Hla in conjunction with Annexin V binding and ApoBrdU Tunel assays examining PBMCs intoxicated with recombinant Hla or infected with USA300, USA300Δ*hla,* USA300Δ*hla* Comp, and USA300Δ*saeR/S* suggest Hla induces programmed cell death of monocytes, B cells, and T cells that results in plasma membrane permeability. Together these findings underscore the importance of Hla during *S. aureus* infection of human tissue and specifically demonstrate Hla activity during USA300 infection triggers programmed cell death of human monocytes, T cells and B cells that leads to plasma membrane permeability.

## Introduction

*Staphylococcus aureus* is a common Gram-positive bacterial pathogen that can produce a wide spectrum of disease in humans, ranging from superficial skin abscesses to invasive life-threatening disease. Emerging antibiotic resistant and hypervirulent strains of *S. aureus* continuously compromise our ability to treat these infections, emphasized by a 2005 survey indicating over 18,000 deaths can be attributed to invasive methicillin resistant *S. aureus* (MRSA) infection in the United States alone [1]. In particular, strains of community-associated MRSA (CA-MRSA) distinct from previously characterized hospital-associated MRSA (HA-MRSA) are a prominent cause of skin and soft-tissue infections and are noted for their enhanced capacity to produce disease in humans [2], [3]. The mechanisms behind the increased virulence observed for CA-MRSA strains remain incompletely defined.

Currently the CA-MRSA strain identified by pulse-field gel electrophoresis (PFGE) as type USA300 is a leading cause of soft-tissue infections in the United States [2], [4]. This strain exhibits a heightened ability to elicit human polymorphonuclear leukocyte (PMN) destruction *in vitro* as well as produce more severe dermonecrotic soft-tissue infections in mice relative to other *S. aureus* strains [5], [6], [7]. Evidence suggests differential expression of core genome-encoded virulence elements is largely responsible for the enhanced virulence observed for USA300 [8], [9], [10]. Indeed, elevated expression of virulence gene regulators *agr*, *saeR/S*, and *sarA* as well as the pore-forming toxin *hla* in USA300 relative to USA400 is thought to contribute to the increased pathogenicity of this strain during rat models of pneumonia [11].

Hla is expressed at higher levels by USA300 during infection of human tissue relative to *in vitro* growth [12] and is largely regulated by the Agr and SaeR/S two-component systems [13], [14], [15], [16]. Recent studies have demonstrated a robust direct regulation of *hla* transcription by SaeR/S and strong attenuation of a USA300 isogenic deletion mutant of this two-component system, suggesting Hla is a primary effector of SaeR/S and a critical component of USA300 virulence [17], [18]. Other research has shown the expression of *hla* directly correlates with *S. aureus* virulence [8], [11], [19] and immunization strategies targeting Hla provide effective protection during murine models of *S. aureus* pneumonia and soft-tissue infection caused by USA300 [5], [20]. However, the exact mechanisms by which Hla promotes *S. aureus* infection are not entirely clear.

To obtain a better understanding of how Hla furthers USA300 pathogenesis, this study examined the influence of this toxin during infection of human blood, purified PMNs, and peripheral blood mononuclear cells (PBMCs) by examining host cell plasma membrane permeability, induction of programmed cell death, and bacterial survival. This investigation demonstrates Hla generated by USA300 triggers programmed cell death in human monocytes, B cells, and T cells that results in plasma membrane permeability while other USA300 components cause immediate plasma membrane permeability to PMNs, monocytes, and B cells but only minimally to T cells. Collectively these findings elucidate the impact of this prominent *S. aureus* toxin on different human blood cell types during infection by USA300.

## Materials and Methods

### Ethics Statement

All human studies were in accordance with an approved protocol by the Montana State University Institutional Review Board. Donors provided written consent to participate in the study. This study (JVK041306) was approved on March 15, 2011.

### Bacterial Strains and Culture

*S. aureus* strains were cultured and harvested as described elsewhere [6], [7], [17], [21]. *S. aureus* pulse-field gel electrophoresis type USA300 (strain LAC) [22] was used to generate USA300Δ*hla* as described previously using oligonucleotides *hla*-Sal1_upstream_fwd (5′-TTCAATGTCGACGGTTAGTCAAAG -3′), hla-HindIII_upstream_rvs (5′-TCGAAATTTTAAAGCTTGATTCAGACTC-3′), hla-SacI_downstream_fwd (5′-TAATGGAGCTCTAATTAATCCGAAATTAATCATG-3′), and hla-BamHI_downstream_rvs (5′-TGTTCAAAACTTTTGGATCCCATTATGTG-3′) and SalI, HindIII, SacI, and BamHI restriction digest [7], [21], [23]. USA300Δ*saeR/S* was generated in previous studies [17]. To construct the USA300Δ*hla* complementing clone, the *hla* gene was PCR amplified from USA300 genomic DNA using oligonucleotides CLM445 (5′-GTTGTTGGATCCCCTTTCTTGAATTAACAATATAC-3′) and CLM446 (5′-GTTGTTGAATTCGCCGAAAAACATCATTTCTG), and cloned into pAH5 using BamHI and EcoRI [24], then transformed into USA300Δ*hla* to generate USA300Δ*hla* Comp. Construction of the USA300Δ*hla* Comp plasmid was confirmed by sequencing using primers CLM445, CLM446, and CLM576 (5′-CCTGTCGCTAATGCCGCAGATTCTG-3′). DNA sequencing was performed at the University of Iowa DNA core facility.

For assays using *S. aureus* supernatants, 20 mL of TSB supplemented with 5% glucose was inoculated with 200 µL USA300 overnight culture, grown for 5 hours with shaking (37°C and 255 rpm), centrifuged (5,000×g, 5 minutes, 4°C), and filtered (0.2 µM). For infection assays, 1 mL of *S. aureus* grown to ME was harvested by centrifugation (5,000×g, 5 minutes, 4°C), washed twice with 1 mL DPBS, and resuspended in 1 mL DPBS (2×10^8^ CFU/mL) prior to inoculation.

### PCR and Western Blot Analysis

PCR was performed using primers for *ACME* (*ACME*_fwd GAGCCAGAAGTACGCGAG, *ACME*-rvs CACGTAACTTGCTAGAACGAG) and *hla* (*hla*_fwd AATGAATCCTGTCGCTAATGCCGC, *hla*_rvs TAAAGGCTGAAGGCCAGGCTAAAC). Western blot analyses for Hla were performed as described [25] on filtered supernatants from *S. aureus* cultures at 5 hours of growth using a rabbit polyclonal anti-Hla antibody (Sigma). Known concentrations of serial diluted recombinant Hla (Toxin Technology) were run on western blot and subject to densitometry analysis to generate a standard curve used to determine concentration of Hla (ng/µL) in 5 hour filtered supernatant of indicated *S. aureus* cultures.

### *S. aureus* Survival in Human Blood

Heparinized venous blood samples from healthy donors were collected in accordance with a protocol approved by the Institutional Review Board for Human Subjects at Montana State University. All donors provided written consent to participate in the study. *S. aureus* was harvested at the mid-exponential (ME) phase of growth (2×10^8^ CFU/mL). Blood (3 mL) was inoculated with 3×10^5^ CFU of *S. aureus* in a 5 mL culture tube and incubated at 37°C with end-over-end rotation (20 rpm). Samples were diluted in sterile deionized water to lyse blood cells and CFUs were determined by plating on tryptic soy agar.

### THP-1 Monocyte Cell Culture and Cytotoxicity Assay

THP1-XBlue-CD14 cells (InvivoGen, San Diego, CA) were maintained around 1×10^6^ cells/mL in growth media (RPMI 1640 medium supplemented with 10% (v/v) endotoxin-free FBS, 200 µg/ml Zeocin and 250 µg/mL G418). THP1 cells (2–4×10^5^ cells) in 100 µL assay medium were combined with 10 µL 5 hour filtered USA300 supernatant or TSB in wells of a flat bottom 96 well plate, then incubated at 37°C for 24 hours. To determine THP1 cell cytotoxicity, 50 µL of sample was analyzed using a Homogeneous Membrane Integrity Assay (Promega) following manufactures protocol. Experiments were performed 3 times with triplicate analysis conducted on each sample per experiment.

### Flow Cytometry

PBMCs and PMNs were isolated from freshly drawn human blood under endotoxin free conditions (<25.0 pg/mL) as described elsewhere [6]. Cell viability and purity of preparations were assessed by flow cytometry (FACSCalibur; BD Biosciences). For flow cytometry analysis, 20 µL of 5 hour *S. aureus* supernatants undiluted or diluted with TSB were added to 1×10^6^ PBMCs or PMNs in 100 µL RPMI with 5 mM HEPES. At indicated times samples were stained with propidium iodide (Invitrogen, Molecular Probes) and FITC or APC mouse anti-human CD3, APC mouse anti-human CD4, APC mouse anti-human CD8, FITC or APC mouse anti-human CD14, or APC mouse anti-human CD19 following the manufacturer’s protocol (BD Pharmingen). 0.1% Triton-X 100 was used as a positive control for cell plasma membrane permeability. Cells were analyzed with a FACSCalibur Flow cytometer (BD Biosciences). The following formula was used to determine % propidium iodide^+^: %Propidium Iodide^+^ = (Mean PI signal_ Experimental_ – Mean PI signal _Blank_)/(Mean PI signal_Triton-X_ – Mean PI signal _Blank_). For infection assays, PMNs or PBMCs (1×10^6^) in 100 µL RPMI with 5 mM HEPES were combined with 50 µL of undiluted or dilute *S. aureus* to obtain the indicated multiplicity of infection (MOI). Samples were centrifuged (500×g, 5 minutes, 4°C), incubated at 37°C for indicated times, and analyzed using flow cytometry as described above. Annexin V-FITC binding assays (Trevigen, Inc) and ApoBrdU Tunel assays (BD Pharmingen) were performed following the manufactures protocol. For assays using recombinant Hla (rHla, Toxin Technology, Inc), 10 µL of rHla or PBS was combined with 20 µL of undiluted supernatant, supernatant diluted with TSB, or TSB alone and 1×10^6^ PBMCs or PMNs in 100 µL RPMI with 5 mM HEPES. The amount of rHla used in these assays is given in hemolytic units (U), where 1 U is the quantity required to generate 50% hemolysis of rabbit blood cells following one hour of intoxication. Samples were incubated at 37°C for 60 minutes then analyzed using flow cytometry as described above.

### Statistical Procedures

Statistical significance was determined with Graphpad Prism (version 4 Graphpad software) using student’s t-test or repeated-measures analysis of variance with Tukey’s post-test for multiple comparisons.

## Results

### Generating an Isogenic Deletion Mutant of *hla* in USA300

**Figure 1:**
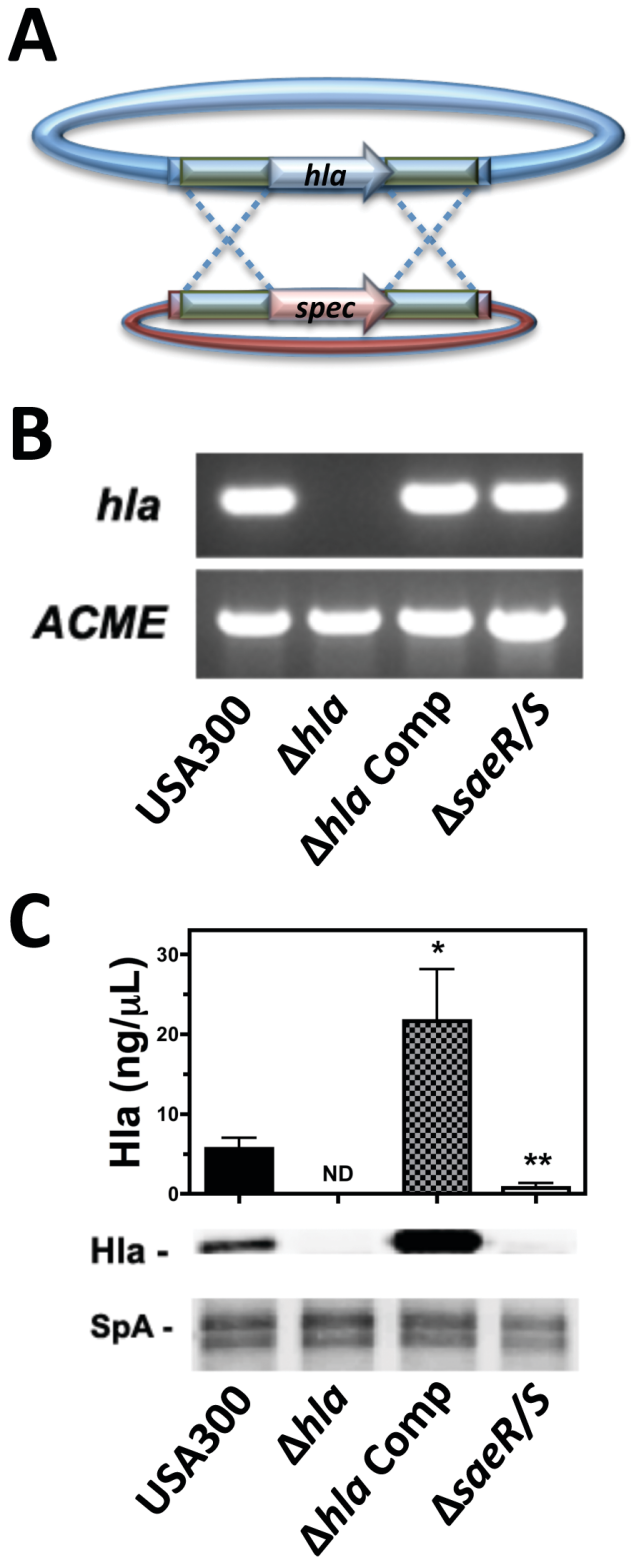
Generation of an isogenic *hla* deletion mutant in USA300. A) Construction of USA300Δ*hla* using allelic gene replacement. B) Colony PCR targeting *hla* and *ACME* and C) Western blot of Hla in 5 hour USA300 supernatants from USA300, USA300Δ*hla* (Δ*hla)*, Δ*hla* supplemented with a complement plasmid encoding *hla* (Δ*hla* Comp), and USA300Δ*saeR/S* (Δ*saeR/S*). Densitometry analysis of Western blots was used to determine Hla concentration (ng/µL) of 5 hour filtered *S. aureus* supernatant using a standardized curve of known rHla concentrations. Data represents 4 replicate experiments. *P<0.05 and **P<0.01 relative to USA300 as determined by paired one-tailed t-test.

To examine the role of *hla* during *S. aureus* pathogenesis, we first developed an isogenic deletion mutant of this toxin in USA300 strain LAC (USA300Δ*hla*) using temperature sensitive allelic replacement (Figure 1A) as previously described [7], [21], [23]. PCR determined the presence of *hla* in USA300, USA300Δ*hla* harboring a plasmid expressing *hla* (USA300Δ*hla* Comp), and an isogenic deletion mutant of *saeR/S* in USA300 (USA300Δ*saeR/S)* but not in USA300Δ*hla* (Figure 1B). Western blot confirmed the deletion of *hla* in USA300Δ*hla* and rescue of production in USA300Δ*hla* Comp (Figure 1C). Hla concentrations where quantified in filtered supernatant from 5 hour cultures of USA300, USA300Δ*hla*, USA300Δ*hla* Comp and in USA300Δ*saeR/S* using densitometry analysis (Figure 1C). Supernatant from USA300 cultures contained 5.634±1.398 ng/µL of Hla in contrast to undetectable concentrations of Hla in supernatant collected from USA300Δ*hla* cultures. Relative to USA300, USA300Δ*hla* Comp supernatant contained a significantly higher concentration of Hla (21.62±6.494 ng/µL) while USA300Δ*saeR/S* contained a significantly lower concentration of Hla (0.7183±0.6513 ng/µL). The diminished concentration of Hla in USA300Δ*saeR/S* supernatant supports *saeR/S*-mediated expression of Hla during *in vitro* growth as suggested by previous studies [17], [26].

### Hla does not Play a Significant Role Inducing PMN Plasma Membrane Permeability

**Figure 2:**
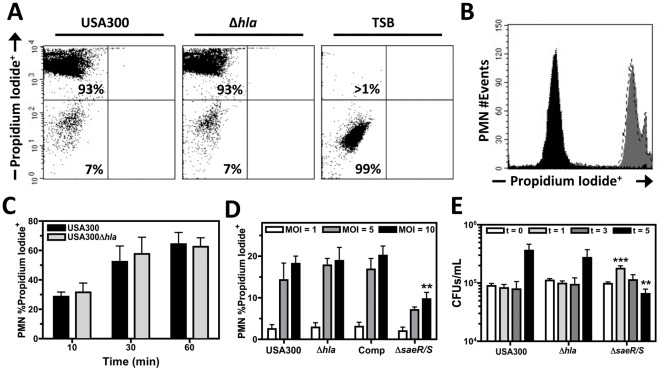
Hla does not significantly influence human PMN plasma membrane integrity or early bacterial survival following USA300 infection of human blood. A) Representative flow cytometry dot plots of human PMNs stained with propidium iodide following intoxication for 60 minutes with 5 hour filtered supernatant from USA300, USA300Δ*hla* or TSB blank. B) Representative Histogram of data in part A with PMNs exposed to supernatant from USA300 indicated by a dashed line, USA300Δ*hla* indicated with grey shading and TSB indicated with black shading. C) Compiled results from part A and B representing 4 separate experiments examining PMN plasma membrane integrity at 10, 30, and 60 minutes post-intoxication with USA300 or USA300Δ*hla* supernatant. D) Flow cytometry analysis of PMNs stained with propidium iodide at 6 hours post-infection with USA300, USA300Δ*hla*, or USA300Δ*saeR/S* at the indicated MOI. Data represents 4 replicate experiments using different blood donors. E) Bacterial counts of USA300, USA300Δ*hla*, and USA300Δ*saeR/S* following exposure to human blood at 0, 1, 3, and 5 hours. For E, data represents 15 replicate experiments from more than 10 separate blood donors. *P<0.05, **P<0.01, and ***P<0.001 relative to USA300 as determined by paired one-tailed t-test.

We examined the ability of USA300 and USA300Δ*hla* to induce plasma membrane permeability of human PMNs (Figure 2). Although rapid plasma membrane permeability was observed for PMNs following intoxication with USA300 supernatants, no significant differences in cytotoxicity could be detected between USA300 and USA300Δ*hla* supernatant (Figure 2A, 2B, and 2C). Assays using USA300, USA300Δ*hla*, USA300Δ*saeR/S*, and USA300Δ*hla* Comp at different MOIs did not reveal any differences in the ability of USA300, USA300Δ*hla*, and USA300Δ*hla* Comp to induce PMN plasma membrane permeability during infection while USA300Δ*saeR/S* caused significantly less PMN plasma membrane permeability at an MOI of 10 (Figure 2D). Under the conditions tested, these data indicate that Hla is not required for PMN lysis by USA300 as previously suggested [16] but are consistent with other investigations demonstrating USA300 produces factors transcriptionally regulated by SaeR/S other than Hla that generate significant PMN and monocyte plasma membrane permeability [17], [25], [27], [28], [29], [30], [31]. In support of these results, no significant difference in survival was noted between USA300 and USA300Δ*hla* during infection of whole human blood while survival of USA300Δ*saeR/*S was significantly increased at 1 hour post-infection and significantly decreased at 5 hours post-infection (Figure 2E). These findings correspond to recent work demonstrating survival of a *saeR/S* isogenic deletion mutant in PFGE type USA400 was significantly reduced relative to the parental wild-type during infection of human blood and upon exposure to PMNs [21]. The inability of USA300Δ*saeR/*S to recognize and appropriately respond to human blood and/or PMN phagocytosis most likely accounts for the differences in bacterial survival observed for USA300Δ*saeR/*S relative to USA300.

### Influence of Hla on Plasma Membrane Permeability Following Intoxication with with USA300, and USA300Δ*hla* Supernatant

**Figure 3:**
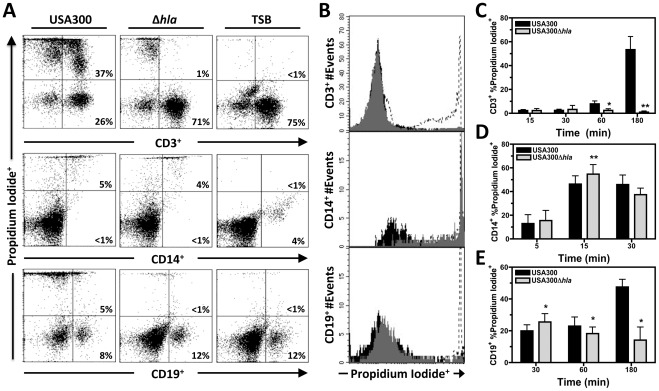
Hla-dependent human T cell and B cell plasma membrane permeablity occurs following 60 minutes exposure to USA300 supernatant. A) Representative flow cytometry dot plots of human PBMCs stained with propidium iodide following intoxication with filtered 5 hour supernatants from USA300 or USA300Δ*hla* diluted to a final concentration of 1∶6 for 30 minutes for CD14^+^ PBMCs or 180 minutes for CD3^+^ and CD19^+^ PBMCs. B) Representative histograms of data in part A with CD3^+^, CD14^+^, and CD19^+^ PBMCs exposed to supernatant from USA300 indicated by a dashed line, supernatant from USA300Δ*hla* indicated with grey shading, and TSB media alone indicated with black shading. Compiled results from at least 4 separate experiments shown in part A using different blood donors for C) CD3^+^, D) CD14^+^ and E) CD19^+^ PBMCs at indicated times following intoxication with *P<0.05, **P<0.01, and ***P<0.001 relative to USA300 as determined by paired one-tailed t-test.

Though Hla did not significantly influence PMN plasma membrane permeability caused by USA300 (Figure 2), other investigations indicate Hla produces pores on a variety of cell types including human lymphocytes [32], [33], [34], [35]. To examine the cytotoxic capacity of Hla expressed by USA300 against different human blood cell types, CD3^+^, CD14^+^, and CD19^+^ PBMCs were examined for plasma membrane permeability at different time points following exposure to USA300 or USA300Δ*hla* supernatant (Figure 3). As with PMNs, rapid plasma membrane permeability was observed for CD14^+^ PBMCs following exposure to USA300 supernatant and accounted for the majority of plasma membrane permeability to these cell types (Figure 3A, 3B, and 3D). Surprisingly, USA300Δ*hla* supernatant caused significantly more CD14^+^ PBMC plasma membrane permeability at 15 minutes than USA300 supernatant, indicating deletion of *hla* enhanced monocyte plasma membrane permeability. In contrast, nearly all CD3^+^ PBMC plasma membrane permeability was caused by Hla and only became apparent after 60 minutes intoxication with USA300 supernatant (Figure 3A, 3B, and 3C). CD19^+^ PBMCs exhibited both rapid plasma membrane permeability that did not require Hla as well as Hla-dependent plasma membrane permeability following 60 minutes of intoxication (Figure 3A, 3B, and 3E). USA300Δ*hla* supernatant caused significantly more CD19^+^ plasma membrane permeability than USA300 supernatant at 30 minutes, again suggesting that mechanisms contributing to plasma membrane permeability that do not require Hla are enhanced in USA300Δ*hla*. Significant differences in CD19^+^ PBMC plasma membrane permeability caused by Hla could only be resolved after 60 minutes of intoxication with USA300 supernatant, similar to the influence of Hla on CD3^+^ PBMCs. Collectively these results indicate that primary human PMNs, monocytes, and B cells but not T cells are susceptible to plasma membrane damage caused by factors other than Hla within 15 minutes exposure to USA300 supernatants while T cell and B cell plasma membrane permeability due to Hla only becomes evident after 60 minutes intoxication.

### Hla is a Major Cause of T Cell Plasma Membrane Permeability during USA300 Infection

**Figure 4:**
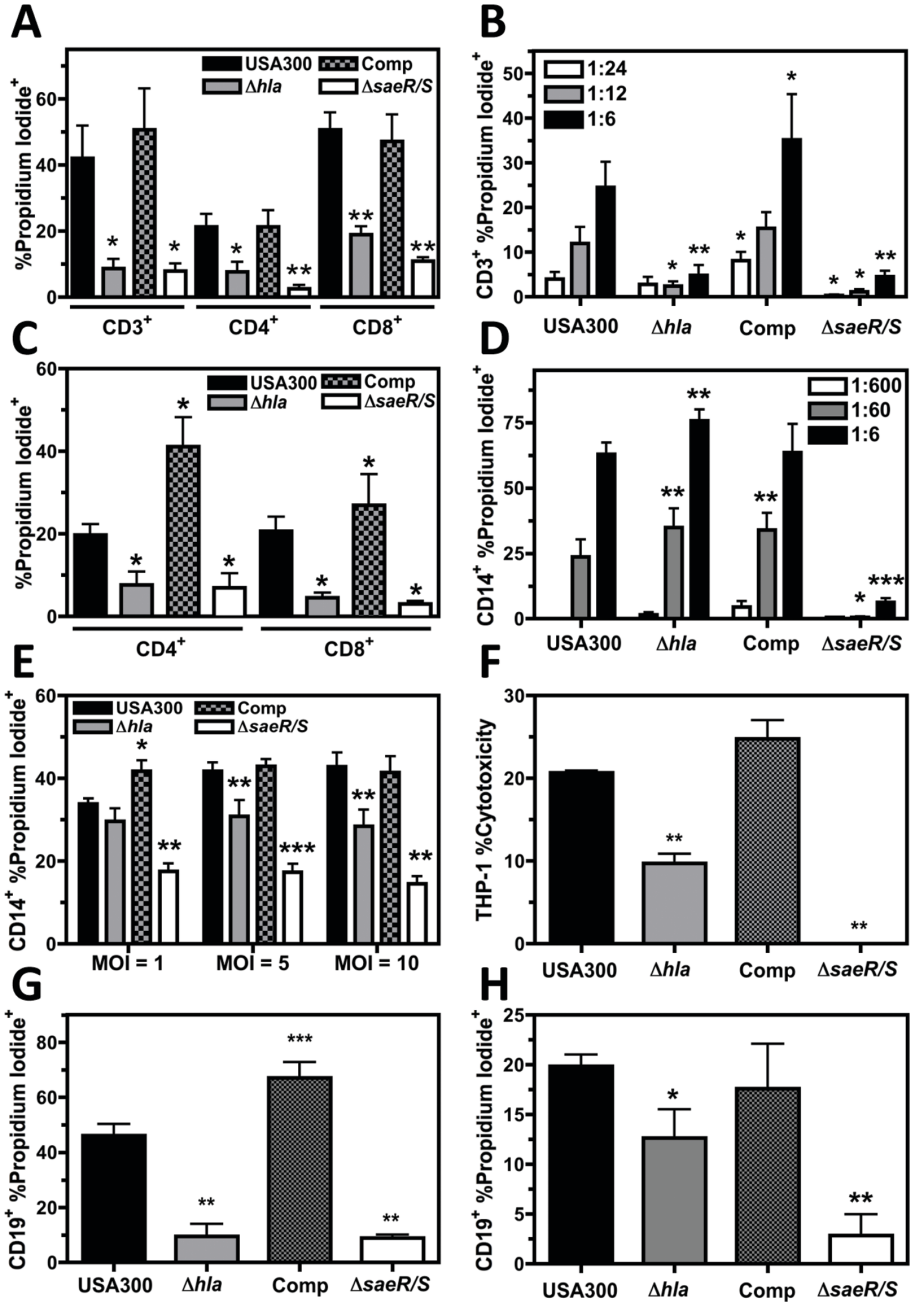
Hla significantly contributes to human T cell, monocyte, and B cell plasma membrane permeability during USA300 infection. A) Compiled flow cytometry analysis of PBMCs intoxicated with USA300, USA300Δ*hla*, USA300Δ*hla* Comp, or USA300Δ*saeR/S* supernatants diluted to a final concentration of 1∶6 for 60 minutes then stained with propidium iodide and anti-CD3, anti-CD4, or anti-CD8. Data represents 4 separate experiments using different blood donors. B) Compiled flow cytometry analysis of PBMCs incubated with *S. aureus* supernatant diluted 1∶6, 1∶12, or 1∶24 for 60 minutes then stained with propidium iodide and anti-CD3. Data represents 5 separate experiments using different blood donors. C) Compiled flow cytometry analysis of CD4^+^ and CD8^+^ PBMCs infected for 6 hours with USA300, USA300Δ*hla*, USA300Δ*hla* Comp, or USA300Δ*saeR/S* at an MOI = 10. Data represents 3 separate experiments using different blood donors. D) Compiled flow cytometry analysis of PBMCs stained with propidium iodide and anti-CD14 following 15 minutes exposure to filtered 5 hour supernatants from USA300, USA300Δ*hla*, USA300Δ*hla* Comp, or USA300Δ*saeR/S* diluted to a final concentration of 1∶6, 1∶60, or 1∶600. Experiments were performed at least 3 times using different blood donors. E) Compiled flow cytometry analysis of CD14^+^ PBMC cytotoxicity at 6 hours post-infection with USA300, USA300Δ*hla*, or USA300Δ*saeR/S* at indicated MOI. Data represents 5 separate experiments using different blood donors. F) Cytotoxicity as determined by LDH release of THP-1 cells intoxicated for 24 hours with USA300, USA300Δ*hla*, USA300Δ*hla* Comp, or USA300Δ*saeR/S* supernatant diluted to a final concentration of 1∶10. Data represents 3 separate experiments. G) Compiled flow cytometry analysis of PBMCs intoxicated for 3 hours at 37°C with USA300, USA300Δ*hla*, USA300Δ*hla* Comp, or USA300Δ*saeR/S* supernatants diluted to a final concentration of 1∶6 then stained with propidium iodide and anti-CD19. Data represents 4 separate experiments using different blood donors. H) Compiled flow cytometry analysis of CD19^+^ PBMCs stained with propidium iodide at 20 hours post-infection with USA300, USA300Δ*hla*, USA300Δ*hla* Comp, or USA300Δ*saeR/S* at an MOI = 10. Data represents 5 separate experiments using different blood donors. *P<0.05, **P<0.01, and ***P<0.001 relative to USA300 as determined by paired one-tailed t-test.

Previous experiments suggested CD3^+^ PBMC plasma membrane damage caused by USA300 is almost entirely Hla-dependent (Figure 3C). To confirm these findings, CD3^+^ PBMC plasma membrane permeability was examined following 60 minutes of intoxication with USA300, USA300Δ*hla,* USA300Δ*hla* comp, or USA300Δ*saeR/S* supernatant (Figure 4A). Significantly less CD3^+^ PI^+^ PBMCs were observed following treatment with USA300Δ*hla* or USA300Δ*saeR/S* supernatant relative to USA300 supernatant while no difference was noted with USA300Δ*hla* comp supernatant, suggesting Hla is an important component of *S. aureus* induced plasma membrane permeability of human T cells. Examination of CD4^+^ PBMCs and CD8^+^ PBMCs (Figure 4A) intoxicated with *S. aureus* supernatant indicated Hla targets both helper T cells (CD4^+^) and cytotoxic T cells (CD8^+^). Using *S. aureus* supernatant diluted by 1∶6, 1∶12, and 1∶24 illustrated concentration-dependent membrane permeability of CD3^+^ PBMCs that was significantly reduced with USA300Δ*hla* or USA300Δ*saeR/S* supernatant relative to USA300 supernatant and significantly enhanced using USA300Δ*hla* Comp supernatant (Figure 4B). PBMCs infected with USA300, USA300Δ*hla,* USA300Δ*hla* comp, or USA300Δ*saeR/S* for 6 hours then stained with PI and either anti-CD4 or anti-CD8 (Figure 4C) also demonstrated that Hla significantly contributes to T cell plasma membrane permeability during infection of PBMCs with USA300. These findings demonstrate Hla is an important virulence gene expressed by USA300 that induces significant T cell plasma membrane permeability.

### Hla Promotes Monocyte Plasma Membrane Permeability

The majority of CD14^+^ PBMC plasma membrane permeability caused by USA300 supernatant was very rapid and occurred in the absence of Hla. However, as Hla-dependent plasma membrane permeability to T cells and B cells required at least 60 minutes to become apparent and mechanisms that did not require Hla caused substantial plasma membrane permeability to the majority CD14^+^ PBMCs within 30 minutes, we further examined CD14^+^ PBMC plasma membrane permeability following intoxication with diluted USA300, USA300Δ*hla,* USA300Δ*hla* comp, and USA300Δ*saeR/S* supernatants (Figure 4D) as well as during infection of PBMCs with these strains (Figure 4E). As observed previously (Figure 3D), USA300Δ*hla* supernatant generated significantly more propidium iodide positive (PI^+^) CD14^+^ PBMCs than USA300 supernatants at 15 minutes post-exposure (Figure 4D). However, infection of PBMCs with USA300, USA300Δ*hla,* USA300Δ*hla* comp, and USA300Δ*saeR/S* at an MOI of 5 and 10 demonstrated Hla significantly enhances CD14^+^ PBMC plasma membrane permeability at 6 hours post-infection (Figure 4E). Further, cultured THP-1 cells incubated for 24 hours with USA300 supernatant containing Hla exhibited significantly increased levels of cytotoxicity as measured by LDH release (Figure 4F). These results indicate rapid CD14^+^ PBMC plasma membrane damage occurs following exposure to USA300 supernatant while Hla-dependent CD14^+^ PBMC plasma membrane permeability occurs at 6 hours post-infection with USA300 and following 24 hours intoxication of THP-1 cells with USA300 supernatant.

### Hla Contributes to B Cell Plasma Membrane Permeability

Rapid CD19^+^ PBMC plasma membrane permeability that did not require Hla as well as Hla-dependent CD19^+^ PBMC plasma membrane permeability was observed following exposure to USA300 supernatant (Figure 3E). To further examine the impact of Hla on B cell plasma membrane permeability caused by USA300, CD19^+^ PBMCs were analyzed with PI staining following 3 hours intoxication with USA300, USA300Δ*hla,* USA300Δ*hla* comp, or USA300Δ*saeR/S* supernatant (Figure 4G). Significantly fewer CD19^+^ PI^+^ PBMCs were observed following exposure to USA300Δ*hla* or USA300Δ*saeR/S* supernatant relative to USA300 supernatant. In contrast, USA300Δ*hla* Comp supernatant induced significantly more plasma membrane permeability than USA300 supernatant. Further, CD19^+^ PBMCs infected with USA300Δ*hla* and USA300Δ*saeR/S* exhibited significantly less plasma membrane permeability at 20 hours when compared to PBMCs infected with USA300 (Figure 4H). These findings demonstrate that Hla significantly contributes to the ability of USA300 to induce human B cell plasma membrane permeability during infection.

### Recombinant Hla Added to USA300Δ*hla* Supernatant Rescues Plasma Membrane Permeability Caused by USA300 Supernatant

**Figure 5:**
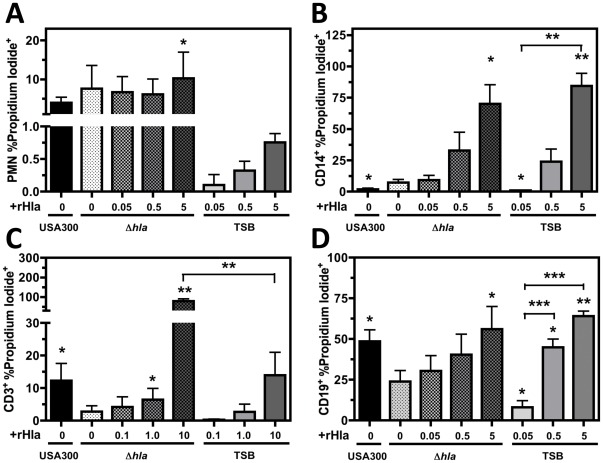
Recombinant Hla alone induces human monocyte, T cell, B cell plasma membrane permeability. Compiled flow cytometry analysis of A) human PMNs intoxicated with indicated amounts of recombinant Hla (rHla) and TSB or *S. aureus* supernatant diluted 1∶60 for 60 minutes, B) human CD14^+^ PBMCs intoxicated with indicated amounts of rHla and TSB or S. *aureus* supernatant diluted 1∶600 for 60 minutes, C) human CD3^+^ PBMCs intoxicated with indicated amounts of rHla and S. *aureus* supernatant diluted 1∶6 for 60 minutes or D) human CD19^+^ PBMCs intoxicated with indicated amounts of rHla and S. *aureus* supernatant diluted 1∶6 for 3 hours. Quantity of rHla added is given in hemolytic units per µL (U/µL). Figures represent at least 4 separate experiments using different blood donors with *P<0.05, **P<0.01, and ***P<0.001 relative to USA300Δ*hla* as determined by paired one-tailed t-test.

To further examine the susceptibility of different human blood cell types to Hla, PMNs and CD14^+^, CD3^+^, or CD19^+^ PBMC plasma membrane permeability was examined following intoxication with increasing concentrations of recombinant Hla (rHla) added to USA300Δ*hla* supernatant or TSB media (Figure 5). Because high concentrations of USA300 supernatant caused robust PMN and CD14^+^ PBMC plasma membrane permeability within 10 minutes, supernatant tested against these cell types was diluted by 1∶60 or 1∶600 with TSB media, respectively. Dilute USA300 supernatant and dilute USA300Δ*hla* supernatant induced similar plasma membrane permeably of PMNs at 1 hour post-intoxication. Plasma membrane permeability caused by dilute USA300Δ*hla* supernatant could only be significantly increased with the addition of 5 U/µL rHla. The addition of 5 U/µL rHla to TSB media increased PMN plasma membrane permeability by approximately 1% (Figure 5A), further suggesting rHla has a minimal effect on PMNs relative to other cytotoxins expressed by USA300. Though dilute USA300 supernatant induced significantly less CD14^+^ PBMC plasma membrane permeability than dilute USA300Δ*hla* supernatant (Figure 5B), addition of 5 U/µL rHla to either USA300Δ*hla* supernatant or TSB media significantly enhanced CD14^+^ PBMC plasma membrane permeability.

In accordance with prior observations, USA300 supernatant exhibited an increased ability to generate both CD3^+^ (Figure 5C) and CD19^+^ (Figure 5D) PBMC plasma membrane permeability relative to USA300Δ*hla* supernatant. Addition of 10 U/µL rHla to USA300Δ*hla* supernatant significantly enhanced CD3^+^ PBMC plasma membrane permeability, supporting prior results indicating this toxin is a primary cause of T cell cytotoxicity induced by USA300. Notably, 10 U/µL rHla combined with USA300Δ*hla* supernatant generated significantly more CD3^+^ PI^+^ PBMCs than 10 U/µL rHla with TSB media alone suggesting Hla works synergistically with other USA300 supernatant components to induce CD3^+^ PBMC plasma membrane permeability. Observed CD19^+^ PBMC plasma membrane permeability following intoxication with USA300Δ*hla* supernatant was significantly increased with the addition of 500 U rHla. Significantly enhanced CD19^+^ PBMC plasma membrane permeability was also observed for either 0.5 or 5 U/µL rHla added to TSB media compared to 0.05 U/µL rHla with TSB media. Collectively these results demonstrate rHla alone can induce significant CD14^+^, CD3^+^, and CD19^+^ PBMC plasma membrane permeability.

### Hla Induces Programmed Cell Death in Monocytes, T Cells and B Cells during Infection by USA300

**Figure 6:**
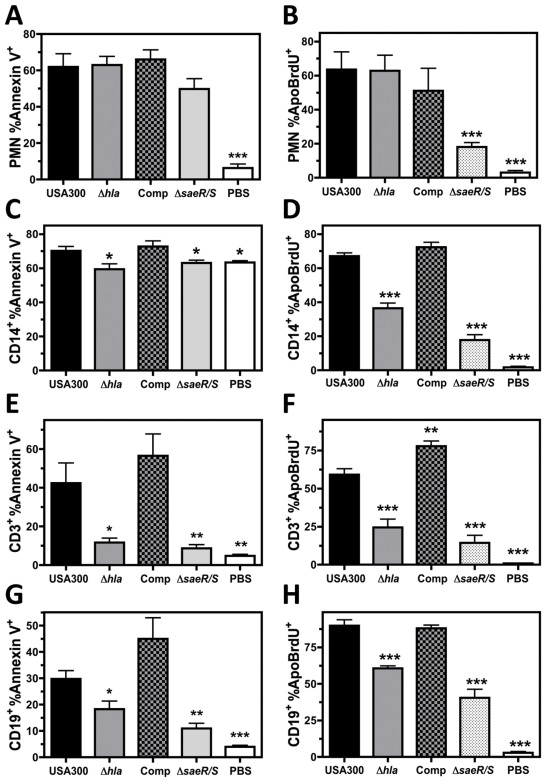
Hla induces programmed cell death in human monocytes, T cells and B cells during USA300 infection. Compiled flow cytometry analysis of human PMNs or PBMCs infected at a MOI of 10 with USA300, USA300Δ*hla*, USA300Δ*hla* Comp, USA300Δ*saeR/S*, or a DPBS control then examined using an Annexin V binding assay at 3 hours (A, C, E, and G) or ApoBrdU Tunel assay at 6 hours (B, D, F, and H). Figures represent 4 separate experiments using different blood donors with *P<0.05, **P<0.01, and ***P<0.001 relative to USA300 as determined by paired one-tailed t-test for A, C, E and G or one-way repeated measures ANOVA with Tukey’s post-test for B, D, F, and H.

A delay in observed Hla-dependent plasma membrane permeability suggested Hla may trigger events that subsequently lead to increased plasma membrane permeability as opposed to directly compromising the plasma membrane integrity to allow passage of PI. To determine if Hla-dependent plasma membrane permeability is the result of events associated with programmed cell death, we performed Annexin V binding and ApoBrdU TUNEL assays on PMNs and PBMCs following infection with USA300, USA300Δ*hla,* USA300Δ*hla* comp, or USA300Δ*saeR/S* (Figure 6). PMNs infected with USA300 exhibited significantly increased Annexin V binding at 3 hours post-infection and significantly enhanced ApoBrdU Tunel staining at 6 hours, indicating phagocytosis of USA300 induces a form of programmed PMN cell necrosis consistent with findings reported by others [36]. Though no differences could be detected in Annexin V binding to PMNs following infection with USA300, USA300Δ*hla,* USA300Δ*hla* comp, or USA300Δ*saeR/S* (Figure 6A), significantly decreased Annexin V binding was observed for CD3^+^, CD14^+^, and CD19^+^ PBMCs at 3 hours post-infection with USA300Δ*hla* and USA300Δ*saeR/S* relative to USA300 (Figure 6C, 6E, and 6G). Constitutively high levels of Annexin V binding were noted for CD14^+^ PBMCs after 3 hours incubation, though significantly decreased Annexin V binding to CD14^+^ PBMCs was observed following intoxication with supernatant from USA300Δ*hla*, USA300Δ*saeR/S*, or PBS control but not following intoxication with USA300Δ*hla* comp supernatant. ApoBrdU Tunel assays further demonstrated a significant decrease of programmed cell death for CD3^+^, CD14^+^, and CD19^+^ PBMCs at 6 hours post-infection with USA300Δ*hla* and USA300Δ*saeR/S* relative to USA300 (Figure 6B, 6F, and 6H). In contrast, a significant decrease in PMN ApoBrdu Tunel staining was observed for infection with USA300Δ*saeR/S* but not USA300Δ*hla*. Together these findings indicate that Hla activity during infection of human blood by USA300 elicits properties characteristic of programmed cell death in human T cells, B cells, and monocytes but not PMNs.

### Intoxication with Recombinant Hla Induced Programmed Cell Death of T Cells, B Cells, and Monocytes

**Figure 7:**
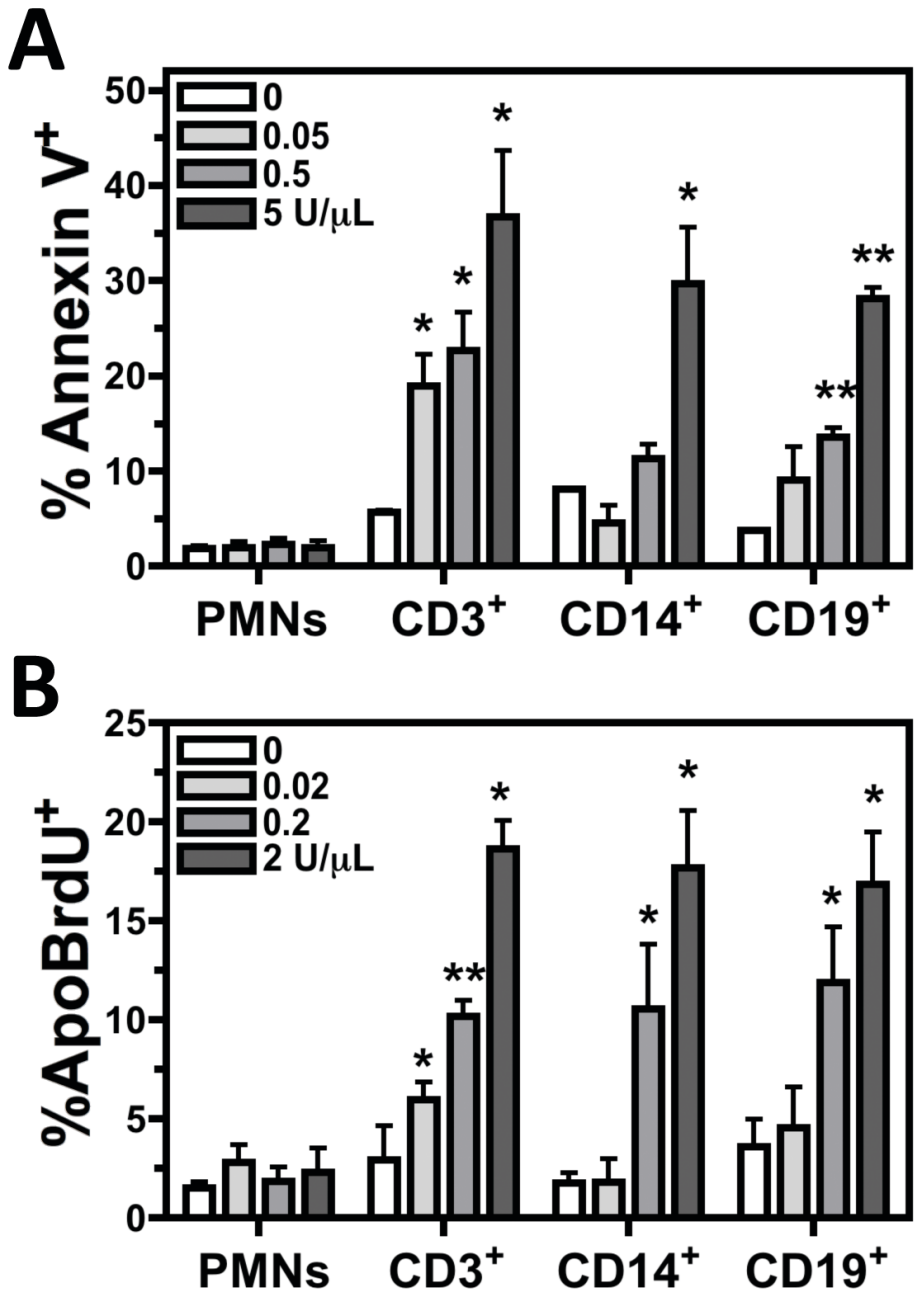
Recombinant Hla alone induces programmed cell death in monocytes, T cells and B cells. Compiled flow cytometry analysis of human PMNs and CD3^+^, CD14^+^, or CD19^+^ PBMCs intoxicated for 60 minutes with increasing concentrations of recombinant Hla then examined using A) Annexin V binding or B) ApoBrdU Tunel assays. Figures represent 3 separate experiments using different blood donors with *P<0.05, **P<0.01, and ***P<0.001 relative to USA300 as determined by one-tailed t-test.

To verify Hla induces programmed cell death of T cells, B cells, and monocytes, Annexin V binding and ApoBrdU Tunel assays were used to examine PMNs and PBMCs intoxicated for 60 minutes with increasing concentrations of rHla (Figure 7). A significant increase of Annexin V binding was observed for CD3^+^, CD14^+^, and CD19^+^ PBMCs following intoxication with 5 U/µL rHla for 60 minutes while no significant difference was noted for Annexin V binding PMNs (Figure 7A). ApoBrdU Tunel assays confirmed a concentration dependent increase in CD3^+^, CD14^+^, and CD19^+^ PBMC programmed cell death in response to intoxication with rHla not observed for PMNs (Figure 7B). Together these results demonstrate rHla alone induces programmed cell death in CD3^+^, CD14^+^, and CD19^+^ PBMCs in a concentration dependent manner while having a minimal influence on PMNs.

## Discussion

Hla is a prominent *S. aureus* virulence factor highly expressed by USA300 and thought to significantly contribute to the virulence of infections caused by this strain [8], [11], [19], yet how this toxin acts to promote pathogenesis is not entirely clear. This investigation demonstrates Hla is the principle component produced by USA300 generating T cell plasma membrane permeability and also significantly compromises B cell and monocyte plasma membrane integrity. In contrast, Hla has a minimal impact on PMN plasma membrane permeability and early bacterial survival during USA300 infection of human blood.

Factors other than Hla in USA300 supernatant contributed to substantial PMN and CD14^+^ PBMC plasma membrane permeability within 15 minutes post-intoxication and caused the majority of observed plasma membrane permeability to these cell types. The observed rapid plasma membrane permeability of PMNs and monocytes in response to USA300 supernatant is likely due to the activity of other pore-forming toxins such as phenol-soluble modulin peptides [27], Panton-Valentine leukocidin [28], [29], leukocidin A/B or G/H [25], [30], and γ-hemolysin [31]. However, while Hla-dependent PMN plasma membrane permeability was not detected during USA300 infection and only minimally detected following intoxication with rHla, Hla significantly influenced CD14^+^ PBMC plasma membrane integrity at 6 hours post-infection with USA300 and rHla alone increased CD14^+^ PBMC plasma membrane permeability in a concentration dependent manner following 60 minutes intoxication. Moreover, USA300 supernatant generated THP-1 cell cytotoxicity that was significantly enhanced by the presence of Hla following 24 hours intoxication. Together these findings indicate Hla produced by USA300 has a significant impact on monocyte plasma membrane permeability during infection but only minimally influences PMN plasma membrane integrity.

Human PBMCs exposed to USA300 supernatant exhibited Hla-dependent T cell and B cell plasma membrane permeability that was only detectable following 60 minutes intoxication. Analysis of PBMCs infected with USA300 relative to USA300Δ*hla* and intoxication of PBMCs with rHla further demonstrated Hla significantly impacts T cell and B cell plasma membrane permeability. Importantly, absence of Hla almost entirely abolished the capacity of USA300 to cause T cell plasma membrane permeability. In contrast, B cells intoxicated with USA300 supernatant exhibited rapid plasma membrane permeability in the absence of Hla as well as plasma membrane permeability following 60 minutes of intoxication that required Hla. These findings indicate Hla is the principle component produced by USA300 generating T cell plasma membrane permeability while B cells are susceptible to Hla in addition to other pore-forming toxins generated by USA300.

How Hla influences the pathogenesis of *S. aureus* disease is not completely understood. *S. aureus* isolates that cause toxic shock syndrome have long been noted for their lack of alpha-toxin production [37], [38], [39], [40], yet experimental evidence clearly demonstrates Hla is critical for USA300 pathogenesis during soft tissue infections and pneumonia caused by *S. aureus* [5], [19], [41], [42]. Diminished expression of Hla by *S. aureus* strains that cause toxic shock syndrome may be at least partially explained by the results in this study showing Hla is the primary factor responsible for T cell plasma membrane permeability caused by *S. aureus*. In the context of toxic shock syndrome, a disease generated by nonspecific binding of TSS Toxin-1 (TSST-1) to the major histocompatibility complex class II molecule of T cells, Hla activity would lead to compromised T cell function that would inhibit the non-specific polyclonal T-cell activation and expansion induced by TSST-1. Thus reduced Hla production by *S. aureus* would be expected in a disease such as toxic shock syndrome that requires T cells to manifest.

As opposed to the rapid plasma membrane permeability that did not require Hla observed for PMNs, monocytes and B cells, Hla-dependent T cell and B cell plasma membrane permeability to PI became apparent only after 60 minutes of intoxication. This observation implies Hla does not immediately induce plasma membrane permeability to PI but instead triggers events that subsequently compromise plasma membrane integrity. Studies using Annexin V binding and ApoBrdU Tunel assays indicated Hla-dependent plasma membrane permeability was associated with induction of features characteristic of programmed cell death in monocytes, T cells, and B cells but not PMNs during infection by USA300. Further analysis of PBMCs and PMNs demonstrated a concentration dependent increase in T cells, B cells, and monocytes undergoing programmed cell death following intoxication with rHla alone that was not observed for PMNs. These results are supported by findings made by others suggesting Hla induces apoptosis in T cells and endothelial cells [34], [43].

This investigation demonstrates that Hla is the principle component produced by USA300 responsible for human T cell plasma membrane permeability and a significant cause of human monocyte and human B cell plasma membrane permeability. Whereas other USA300 cytotoxic factors immediately generate plasma membrane permeability to PI, Hla is shown to induce programmed cell death in susceptible cell types that subsequently compromises plasma membrane integrity. Together these findings demonstrate that Hla has a significant impact on human immune cell integrity and function during USA300 infection and may partially explain why both passive immunization with Hla-specific anti-serum as well as active immunization with non-toxigenic recombinant Hla has proven effective in murine models of soft tissue infection and pneumonia [5], [20] given the numerous unsuccessful past attempts at developing a suitable vaccine to protect against *S. aureus* infection [44], [45], [46].
